# Enteric Pathogens and Coinfections in Foals with and without Diarrhea

**DOI:** 10.1155/2016/1512690

**Published:** 2016-12-27

**Authors:** Giovane Olivo, Thays Mizuki Lucas, Alexandre Secorun Borges, Rodrigo Otávio Silveira Silva, Francisco Carlos Faria Lobato, Amanda Keller Siqueira, Domingos da Silva Leite, Paulo Eduardo Brandão, Fábio Gregori, José Paes de Oliveira-Filho, Shinji Takai, Márcio Garcia Ribeiro

**Affiliations:** ^1^Department of Veterinary Clinical Sciences, Universidade Estadual Paulista (UNESP), Botucatu, SP, Brazil; ^2^Department of Veterinary Hygiene and Public Health, Universidade Estadual Paulista (UNESP), Botucatu, SP, Brazil; ^3^Department of Preventive Veterinary Medicine, Federal University of Minas Gerais (UFMG), Belo Horizonte, MG, Brazil; ^4^Department of Genetics, Evolution and Bioagents, Campinas State University (Unicamp), Campinas, SP, Brazil; ^5^Department of Preventive Veterinary Medicine, University of São Paulo, São Paulo, SP, Brazil; ^6^Department of Animal Hygiene, Kitasato University, Towada, Aomori, Japan

## Abstract

Diarrhea is a major clinical problem affecting foals up to 3 months of age. The aim of this study was to identify enteric microorganisms involved in monoinfections and coinfections and the associated virulence factors in healthy and diarrheic foals. Diarrheic (D) (*n* = 56) and nondiarrheic (ND) foals (*n* = 60) up to three months of age were studied. Fecal samples were analyzed for identification of infectious agents (microbiological culturing, molecular techniques, and microscopic analyses).* Escherichia coli fimH* (30% versus 25%),* Salmonella* spp. (25% versus 7%),* Strongyloides westeri* (25% versus 25%),* Clostridium perfringens* type A (21% versus 10%),* E. coli ag43* (20% versus 35%),* Strongylus* (11% versus 18%), and* vapA*-positive* Rhodococcus equi* (5% versus 2%) were the most frequent enteric pathogens detected in D and ND foals, respectively. The frequency of toxin A-positive* C. perfringens* was significantly increased in the D (*p* = 0.033) compared with the ND animals.* R. equi* strains harboring virulent plasmids were also identified (VapA 85-kb type I and VapA 87-kb type I) in D and ND foals. Coinfections were observed in 46% of the D and 33% of the ND foals. Our results demonstrate the great diversity of enteric pathogens, virulence factors, and coinfections involved in enteric infections of foals.

## 1. Introduction

Diarrhea is one the most common causes of mortality of neonatal foals, which results in economic losses worldwide [1, 2]. Until 6 months of life, up to 20% of foals have been reported to suffer from diarrhea caused by infectious agents [3]. The etiology of diarrhea in neonatal foals is complex and involves infectious agents, management, and facilities, as well as nutritional, environmental, and physiological conditions [3–5].

Infectious causes of foal diarrhea predominantly include bacteria, viruses, and parasitic agents [5–7]. Some studies of diarrhea in neonatal foals have focused on only one pathogen [2, 8–11] although it is usually caused by diverse combinations of enteric infections [1, 5, 7, 12, 13]. The understanding of the prevalence and complexity of major pathogens associated with enteric infections in neonatal foals is limited [14]. Comprehensive studies have highlighted the impacts of enteric coinfections in foals with diarrhea and have identified major virulence factors of some pathogens in these infections [1, 5, 7, 13]. However, few studies have evaluated the normal microbial population of the equine intestine, particularly in foals [15–17]. Nondiarrheic foals have been suggested to be potential reservoirs of enteric pathogens, including agents with zoonotic potential [13, 18]. The aim of this study was to identify enteric microorganisms involved in monoinfections and coinfections and the associated virulence factors in healthy and diarrheic foals up to three months of age and to identify dehydration, alterations in the complete blood count (CBC), and electrolyte abnormalities.

## 2. Materials and Methods

### 2.1. Study Design

Fecal samples were collected from 56 diarrheic and 60 nondiarrheic foals of different breeds up to 90 days of age and divided into three groups according to age for bacteriological, virological, and parasitological tests. Monoinfections and coinfections with different enteric pathogens were investigated, as well as selected virulence factors and toxin production in* Escherichia coli*,* Clostridium* sp., and* Rhodococcus equi* isolates. Clinical abnormalities and hematological and blood gas parameters were also assessed in the diarrheic foals. This study was carried out from July 2011 to October 2012 at horse farms in the central region of the State of São Paulo, Brazil.

### 2.2. Selection of Farms and Animals

A cross-sectional study was carried out at 15 horse farms (50–150 horses per farm) (Table 1). All foals with diarrhea identified at each farm were included in the study. Samples were collected from each foal only once. Brief clinical histories were also obtained from the diarrheic and nondiarrheic animals. The exclusion criteria were as follows: (1) foals undergoing antimicrobial therapy and (2) foals older than 90 days of age. An average of 7.5 foals per farm (1 to 28) was used. All of the farms were similar in terms of the general management of the horses, animal hygiene, and facilities, and they all had histories of diarrhea in foals. None of the equines had been vaccinated against rotavirus or other enteric pathogens.

All of the procedures followed in this study were approved by the Animal Use and Ethics Committee (CEUA), Brazil (CEUA–protocol 155/2011).

### 2.3. Clinical Examination

Prior to obtaining fecal samples, all foals underwent clinical examination [19–21]. The foals with diarrhea had abnormal fecal consistency and color, and clinical signs (anorexia, depression, and/or weakness) lasting for more than 24 hours. All foals with and without diarrhea were nursed and were thus considered to have ingested colostrum within the first 24 hours of life (nursing was observed). Dehydration rates were classified at clinical examination as discrete (6%), moderate (8 or 10%), and severe (≥12%). Dehydration was estimated based on several physical parameters (skin elasticity, mucous membrane color, sunken eyes/enophthalmia, capillary refill time, and heart rate) [4, 21].

### 2.4. Groups

The 116 foals were divided into diarrheic and nondiarrheic groups. They were then subdivided into the following three groups according to the age: GI (0–30 days of age); GII (31–60 days); and GIII (61–90 days).

### 2.5. Fecal Samples

Fresh fecal material was collected directly from the rectum of each animal and was then stored at 4–8°C and transported to the laboratory. In addition, aliquots of fecal samples were kept frozen (−80°C) in the laboratory until analyses with different methods.

### 2.6. Identification of* Escherichia coli*,* Rhodococcus equi*,* Clostridium perfringens*,* Clostridium difficile*, and* Salmonella* spp. 

For the culturing of* Escherichia coli*, fecal material was plated on defibrinated sheep blood agar and MacConkey agar and evaluated at 24, 48, and 72 hours [22]. In addition, microbiological culturing of the feces was performed using CAZ-NB selective media for* Rhodococcus equi* isolation [23].

For the isolation of* C. perfringens* and* C. difficile*, fecal material was diluted. Aliquots were plated in sulfite polymyxin sulfadiazine agar and in taurocholate cycloserine cefoxitin fructose agar and incubated anaerobically. Suspected* Clostridium* colonies were classified using conventional phenotypic methods [24] and were then analyzed by PCR for detection of the major* C. perfringens* toxin genes (alpha, epsilon, beta, and iota) and beta-2, NetB-, and enterotoxin-encoding genes [25, 26]. For* C. difficile*, a multiplex PCR for a housekeeping gene (*tpi*), toxins A (*tcdA*) and B (*tcdB*), and a binary toxin gene (*cdtB*) was performed on all suspected colonies [27].

For* Salmonella* detection, samples were inoculated into specifics Rappaport-Vassiliadis and Tetrathionate broth. Each broth culture was plated in xylose-lysine-deoxycholate agar and in bismuth sulfite agar and was then incubated. Suspected* Salmonella* sp. colonies were inoculated in triple sugar iron and lysine iron agar and classified using conventional biochemical tests [24]. A commercial agglutination test using polyvalent anti-*Salmonella* serum (Probac™) and serotype identification were also carried out [22].

### 2.7. Molecular Detection of* E. coli* and Virulence Factors

*E. coli* strains were cultured overnight in BHI (Brain Heart Infusion) and were then inoculated in trypticase soy agar for confluent growth overnight, followed by DNA extraction. The sequences of the primers used, predicted sizes of the amplified products, and specific annealing temperatures have been previously described [28]. The following groups of genes were analyzed by PCR:* papC* and* papG* alleles (P fimbria),* sfaC*/*D* (S fimbria),* afaB*/*C*,* saa*,* iucD*,* cnf-1*,* cnf-2*,* hly*,* vt1*,* vt2*,* sta*,* stb*,* ipaH*, and* eae*,* eaf*, and* bfp*.

### 2.8. Virulence of* R. equi*

Plasmid DNA was isolated using the alkaline lysis method with several modifications [29]. The target DNA for PCR amplification was based on reported vapA (15- to 17-kDa antigen) and vapB (20-kDa antigen) gene sequences (GenBank accession numbers D212361 and D44469). Plasmid DNA was digested with the restriction endonucleases* Eco*RI,* Eco*T22I, and* Hind*III [30, 31]. Then, the plasmid samples were separated by electrophoresis and examined under UV light. PCR amplification was performed as previously described [30].

### 2.9. Cytotoxicity Assay (CTA) of* C. difficile*

*C. difficile* A/B toxins were investigated using Vero cells (Vero ATCC CCL 81) [32]. Fecal samples were diluted in phosphate-buffered saline (PBS) and centrifuged. The supernatants were filtered and diluted twofold to 1 : 1,024, and then serial dilutions and parallel samples containing* Clostridium sordellii* antitoxin were added to Vero cell monolayers. The cells were examined after incubation. A sample was considered positive in CTA if at least 90% of cells were round and if the effect was neutralized by the antitoxin at the same dilution in the parallel sample [11].

### 2.10. Rotavirus and Coronavirus Detection

RNA was extracted from fecal samples using TRIzol™ reagent (Invitrogen™), following the manufacturer's instructions, and the phenol/chloroform method [33]. Samples were tested for the presence of coronavirus using a* Betacoronavirus*-1-specific RT-PCR assay targeting the RNA-dependent RNA-polymerase gene (RdRp) [34]. The BCoV Kakegawa strain and PBS were used as positive and negative controls, respectively. The samples were also analyzed for the presence of rotavirus by polyacrylamide gel electrophoresis (PAGE) [35]. The NCDV group A rotavirus strain was used as a positive control.

### 2.11. *Lawsonia intracellularis*,* Cryptosporidium parvum*, and* Giardia duodenalis* DNA Detection

DNA was extracted from fecal samples using a Qiagen DNA Stool Mini kit (Qiagen™). Nested PCR amplification was performed using the set of primers previously described for detection of* Cryptosporidium* spp. [36, 37],* G. duodenalis* [38, 39], and* L. intracellularis* DNA [8, 40], with some modifications. Nested PCR products from the genera* Cryptosporidium* and* Giardia* with the expected sizes were analyzed by automated direct sequencing using a 3500 Genetic Analyzer (Applied Biosystems, Foster City, CA) and BigDye™ Terminator v3.1 Cycle Sequencing Kit.

### 2.12. Parasitological Examination

To identify intestinal parasites (helminthes), fresh fecal samples were assessed by flotation in zinc sulfate, followed by microscopic examination [41] in a McMaster counting chamber.

### 2.13. Analysis of Blood from Diarrheic Foals

Blood counts were performed using a hematological counter (Poch 100iV Diff, Roche®). Hematocrit (Htc) was measured using the microhematocrit method. Leukocyte counts were performed using 100 cells, along with evaluations of erythrocyte, leukocyte, and platelet morphologies, in blood smears stained using quick Panotic dye (LaborClin®). Analysis of venous blood [blood pH, Htc, hemoglobin (Hb), partial pressure of CO_2_ (PCO_2_), partial pressure of O_2_ (PO_2_), total CO_2_ concentration (tCO_2_), oxygen saturation (SO_2_), base excess, and HCO_3_, Na, K, and ionized calcium (iCa) concentrations] was performed with I-STAT EG7+ Cartridges (Abbott™, East Windsor, NJ, USA).

### 2.14. Statistical Analysis

The chi-square or Fisher's exact test was used to compare the frequencies of the different enteric pathogens between the diarrheic and nondiarrheic foals and to compare the presence of different virulence factors and production of toxins by isolates from these two groups of foals. Statistical analyses were conducted using SAS/STAT [27], and the statistical significance level was set at 0.05.

## 3. Results

### 3.1. Clinical Signs of Dehydration and Diarrhea

The diarrheic and nondiarrheic sampled foals were distributed into the following three age groups: GI (0–30 days), with 19 diarrheic and 10 nondiarrheic foals; GII (31–60 days), with 17 diarrheic and 13 nondiarrheic foals; and GIII (61–90 days), with 20 diarrheic and 37 nondiarrheic foals. Signs of dehydration were observed exclusively in the diarrheic foals, among which 48.2% (27/56), 16% (9/56), and 3.6% (2/56) showed discrete, moderate, and severe dehydration, respectively.

Gastrointestinal tract auscultation resulted in the identification of increased intestinal sounds in the diarrheic foals, as well as a high frequency of defecation in 95% (*n* = 53/56) of the animals.

### 3.2. Hematological Findings and Blood Gas Parameters

Hematological evaluation of the diarrheic foals revealed that 46.9% (15/32) had an increased plasmatic fibrinogen level. In addition, leukocytosis was observed in 15.5% (7/45) of the diarrheic foals, and neutrophilia was detected in 22% (10/45). Lymphocytosis was observed in 37% (21/56) of the animals.

Blood gas examination revealed that 16% (9/56) of the foals had low pH (range 7.3 ± 0.1) and blood bicarbonate levels, characteristic of metabolic acidosis. Electrolyte imbalances were evident in the diarrheic foals, with 5.3% (3/56) exhibiting hypocalcemia, 66% (37/56) displaying hyponatremia (133.4 mmol/L ± 3.3), and 39% (22/56) exhibiting hypokalemia (3.1 mmol/L ± 0.7) (Table 2).

No associations were observed between the hematological or blood gas parameters and the enteric agents assessed or the mortality rate among the foals. In contrast, a tendency toward abnormal values of the hematological and blood gas parameters was observed in the foals with severe dehydration and signs of diarrhea.

### 3.3. Enteric Pathogens

The frequencies of enteric pathogens of bacterial, parasitic, and viral origins, as well as the selected virulence factors detected in the diarrheic and nondiarrheic foals sampled, are summarized in Table 3.

The most frequent enteric pathogens identified in the diarrheic foals were as follows: 30.3% (17/56)* E. coli fimH*, 25% (14/56)* Salmonella* spp., 25% (14/56)* Strongyloides*, 21.4% (12/56)* Clostridium perfringens* type A, 19.6% (11/56)* E. coli ag43*, 10% (6/56)* Strongylus*, and 5% (3/56)* R. equi vapA*. In contrast, the most frequent enteric pathogens found in the nondiarrheic foals were as follows: 35% (21/60)* E. coli ag43*, 25% (15/60)* E. coli fimH*, 25% (15/60)* Strongyloides westeri*, 18% (11/60)* Strongylus*, 10% (6/60)* C. perfringens* type A, and 2% (1/60)* R. equi vapA*.

*C. perfringens* type A was isolated from 21% and 10% of the diarrheic and nondiarrheic foals, respectively. Four of these strains were positive for the beta-2 toxin-encoding gene (*cpb2*), including three strains from apparently healthy animals and one from a diarrheic foal. None of the isolates were positive for NetB- or enterotoxin-encoding genes. The isolation of* C. perfringens* was significantly associated with the presence of diarrhea (*p* = 0.033). Among the 12 diarrheic foals that tested positive for* C. perfringens* type A, three (25%) also tested positive for another enteropathogen.* C. perfringens* type C was not detected in this study.

*C. difficile* infection was confirmed in only one diarrheic foal in this study. This animal, which was 19 days old, tested positive for the A/B toxins, and a toxigenic* C. difficile* strain (A+B+CDT−) was isolated. Three other strains of* C. difficile* were isolated in this study, including two nontoxigenic strains (A−B−CDT−) that were both isolated from nondiarrheic animals (GI), and a toxigenic strain (A+B+CDT−) that was isolated from a one-day-old foal that tested negative for the A/B toxins.

Seven different serotypes of the* Salmonella* genus were identified. Among them, fourteen were isolated from the diarrheic foals, with a predominance of* Salmonella* Infantis,* Salmonella* Typhimurium, and* Salmonella* Saintpaul. Among the nondiarrheic animals, four serotypes were identified, namely,* Salmonella* Infantis,* Salmonella* Saintpaul,* Salmonella* Typhimurium, and* Salmonella* Newport.* Salmonella* serotypes were identified in single and combined infections in 3 and 11 diarrheic foals, respectively. In contrast, only four nondiarrheic foals tested positive for* Salmonella*, which was present together with other enteric pathogens in all of the animals.

*Salmonella* Saintpaul (67% versus 33%),* S.* Panama (100% versus 0%),* S.* Infantis (83% versus 17%),* S.* Typhimurium (75% versus 25%),* S. enterica* subsp.* enterica* (100% versus 0%),* S.* Muenchen (100% versus 0%),* C*.* perfringens* toxin A (67% versus 33%), and* R. equi* vapA (75% versus 25%) exhibited higher prevalence rates in the diarrheic foals than in the nondiarrheic foals.

Seventeen and 15 strains of* Escherichia coli* were isolated from the feces of the diarrheic and nondiarrheic foals, respectively. The virulence factors* fimH* and a*g43* were detected in 32 isolates, of which 11 were obtained from diarrheic animals. The* papC* gene was detected in 4 diarrheic and 2 nondiarrheic foals. Coinfections of* E. coli* with specific virulent factors and* Salmonella* serotypes in the diarrheic foals were observed as follows:* Salmonella* Infantis,* E. coli fimH* and* papC*;* Salmonella* Newport,* E. coli fimH* and* ag43* (Table 6);* Salmonella* Infantis,* E. coli fimH* and* ag43*; and* Salmonella* Infantis,* E. coli fimH*. Among the nondiarrheic foals, the following coinfections were observed:* Salmonella typhimurium*,* E. coli* ag43 and fimH;* Salmonella saintpaul* and* E. coli* ag43. Only one foal that tested positive for* E. coli* showed clinical signs of sepsis.

Nine samples from all foals tested positive for* R. equi*; three strains were identified in the diarrheic foals, and six were detected in the nondiarrheic foals. Virulence plasmid analysis of the nine strains revealed that one VapA 85-kb type I isolate and one VapA 87-kb type I isolate were present in two diarrheic animals in group I. One diarrheic animal in group II was positive for VapA 85-kb type I, whereas a nondiarrheic animal in the same group was positive for VapA 87-kb type I. The remaining five* R. equi* isolates identified in the nondiarrheic animals were considered avirulent (absence of the* vapA* and* vapB* genes).

Nonconventional primary agents of diarrhea in foals, such as* Hafnia alvei*,* Proteus mirabilis*,* Proteus vulgaris*,* Enterobacter agglomerans*,* Providencia rettgeri*, and* Citrobacter* spp., were identified in single isolates (without coinfection with other major enteric pathogens), despite their low frequencies (Table 4).* Serratia rubidaea*,* Edwardsiella agglomerans*,* Edwardsiella tarda*,* Citrobacter* spp., and* Hafnia alvei* were recovered in pure cultures at low frequencies in the nondiarrheic animals (without coinfection with enteric pathogens) (Table 4).

None of the fecal samples tested positive for* Lawsonia intracellularis*. Five (4%) animals up to 90 days of age tested positive for* Cryptosporidium parvum* (3 foals with diarrhea and 2 nondiarrheic foals). One diarrheic foal and one nondiarrheic foal presented* C. parvum* as the only detected pathogen.

Sequence analysis of the genetic material resulted in detection of* Giardia duodenalis* in only two (2%) foals in group II, including one diarrheic and one nondiarrheic animal.

Helminths were identified at relatively similar frequencies between the diarrheic and nondiarrheic foals up to 90 days of age, with the highest frequency observed in group III. No helminth was detected as a single pathogen in the diarrheic foals; rather, these enteric parasites were identified in combined infections among the diarrheic samples.

Only two foals tested positive for coronavirus (one diarrheic foal in group I and one nondiarrheic foal in group III). Rotavirus was not detected in the studied samples.

### 3.4. Co-Infections and Mortality of Foals

The distribution of enteric pathogens identified alone or in combination in the diarrheic and nondiarrheic foals up to 90 days of age is shown in Table 4. A total of 87% (49/56; *p* < 0.27) of the fecal samples from the diarrheic foals tested positive for enteric pathogens, in addition to 80% (48/60; *p* < 0.27) of those from the nondiarrheic animals. Among the diarrheic foals, 46% had coinfections, and 41% had monoinfections. In contrast, 47% of the nondiarrheic foals had monoinfections, and 33% had coinfections (*p* < 0.29) (Table 4). The most common coinfections involved* Strongyloides westeri*,* Strongylus*, and* Salmonella* spp.; combined infections with VapA-positive* R. equi* and* C. perfringens* toxin A were also common. However, no significant difference was observed between the most common enteric pathogens detected in the mono- versus coinfections (Table 5).

Eight foals died during the study (six within diarrhea group).* Salmonella* was detected in 7 out of 8 of the dead animals. The following coinfections were detected in the dead animals: (1)* E. coli*,* Clostridium perfringens* type A,* Salmonella* Panama,* Strongyloides westeri*, and Strongylus; (2)* E. coli fimH*,* E. coli papC*,* Salmonella* Infantis, and* Strongyloides*; (3)* Salmonella* Typhimurium and* Strongyloides westeri*; (4)* Salmonella enterica* subsp.* enterica* and* Strongyloides westeri*; and (5)* Salmonella* Muenchen and* Strongyloides westeri* (Table 6). Only one dead foal tested negative for all enteric pathogens. With regard to the two nondiarrheic foals that died, one was positive for* E. coli* and* Salmonella* Infantis, while the other was positive for* E. coli fimH*,* E. coli ag43*,* Salmonella* Newport, and* Strongyloides westeri* (Table 6). The relationships between the mortality rate and age in the diarrheic and nondiarrheic foals are shown in Table 6.

## 4. Discussion

Clinically, increased intestinal motility and signs of dehydration were observed in the diarrheic foals, similar to previous studies [20, 42]. The mean clinical parameters (temperature and cardiac and respiratory rates) and hematological findings (leukocytes, lymphocytes, neutrophils counts, total protein, and fibrinogen concentrations) did not significantly differ between the diarrheic and nondiarrheic foals and reference values. In contrast, increases in the total numbers of leukocytes, neutrophils, and lymphocytes and fibrinogen concentration were observed in the foals with profuse diarrhea, in agreement with a previous study [1].

Blood gas analysis of 56 diarrheic foals revealed that 16% had metabolic acidosis [19, 43]. Diarrheic foals have low levels of sodium, potassium, and calcium due to electrolyte loss caused by the diarrhea [44]. Despite the lack of significant associations among the specific enteric pathogens assessed, the diarrheic foals, mainly the severe diarrheic foals, exhibited a tendency toward abnormal blood gas levels. These results indicate that the use of manual blood gas analysis equipment may be important for the diagnosis of severe clinical cases and may enable early application of critical care therapy.

The etiology of infectious diarrhea in neonatal foals is complex [3–5]. In fact, a wide variety of agents of bacterial, viral, and parasitic origins and their virulence factors were detected in the current study.* C. perfringens* and* C. difficile* are considered frequent causes of colitis in humans [18] and livestock [4]. Enteric infections are prevalent in adult horses, neonatal foals, foals with antimicrobial-associated diarrhea, and equines affected by nosocomial outbreaks in veterinary hospitals [44–46].* C. perfringens* type C, commonly associated with necrotic enteritis in neonatal foals [47], was not found in the present study. On the other hand, an association between diarrhea and isolation of* C. perfringens* type A was observed, as in previous studies [1, 11, 48, 49].

Four of 18 (22%)* C. perfringens* isolates were positive for the beta-2 toxin gene (*cpb2*), although only one* cpb2*+ was isolated from a diarrheic animal. Few studies have examined the role of beta-2 toxin as an additional virulence factor in* C. perfringens* infection in equines, and almost all of them have exclusively examined adult horses [50, 51]. Thus, the importance of beta-2 toxin in foals remains controversial [11].

A toxigenic* C. difficile* strain (A+B+CDT−) was isolated from one diarrheic foal in this study; this was the only confirmed case of* C. difficile* infection in this study. However,* C. difficile* strains were isolated from three other animals. Among them, two strains were nontoxigenic (all from nondiarrheic animals), and one was toxigenic but was negative for the A/B toxins. The lack of detection of the A/B toxins in these three animals may indicate that they are asymptomatic carriers, which may be a common occurrence among foals [11, 45].

Salmonellosis is certainly one of the most important zoonoses worldwide [18]. In equines, it has been identified as a prevalent causative agent of enteric and systemic infections [52]. A total of 18* Salmonella* strains were isolated from foals up to 90 days of age in the current study. This result is consistent with those of other studies demonstrating that this pathogen is more prevalent in animals between 1 and 3 months of age [13, 52]. In the present study, 25% (14/56) of the* Salmonella* strains were isolated from diarrheic foals; this is considered a high frequency and is in agreement with the results of similar studies [13, 53].* Salmonella* Typhimurium appears to be the most commonly reported pathogenic serotype in equines of different ages [46]. Over the last decades,* Salmonella* Typhimurium, Newport, Anatum, and Agona were the most frequent serotypes reported in the USA [52]. In addition,* Salmonella* Typhimurium, Newport and Glostrup were the main serotypes detected among diarrheic horses in Brazil [54]. In the current study,* Salmonella* Infantis, Typhimurium, and Saintpaul were the most prevalent serotypes recovered from the diarrheic and nondiarrheic foals. In addition, higher mortality of the* Salmonella*-infected foals compared with the non-*Salmonella*-infected foals was observed in the present study.

*E. coli* was isolated alone or together with other pathogens at a high frequency in both the diarrheic or nondiarrheic foals. This result is not surprising because this organism is part of the normal enteric microflora of livestock [4]. Despite the recognized pathogenicity of* E. coli* to humans [18] and livestock [4] the major virulence factors of* E. coli* involved in enteric infections of foals remain unclear [4]. Selected virulence factors of* E. coli* were assessed in 99* E. coli* strains from the foals; three strains from diarrheic foals were positive for Shiga-like toxin genes and another isolate was positive for STb and LT, whereas one strain from a nondiarrheic foal was positive for STb. In addition, eight isolates from diarrheic foals were positive for the* eae* gene. These findings demonstrate the presence of potentially virulent strains [40]. In the current study, the absence of F4 (k88), F5 (k99), and F41 fimbriae and LT, STa, and STb enterotoxins among the* E. coli* strains isolated from diarrheic and nondiarrheic foals indicates that this organism has little influence on the pathogenicity of enteric infections in neonatal foals, despite the fact that F4 and F5 fimbriae are classically involved in enterotoxigenic infections in calves and pigs and have been identified sporadically [2] or at low levels in enteric infections of foals [40]. Other virulence factors related to enteric and/or extraenteric infections in domestic animals, such as the* cnf-1* and* cnf-2* cytotoxins,* papG*,* sfaC*/*D*, and* afaB*/*C* fimbriae and* iucD* (aerobactin) and* hly* genes [28], were not detected in the 32* E. coli* strains assessed in this study, indicating that* E. coli* may not be a typical primary agent of colitis or enterocolitis in foals [17]. Despite the apparent lack of an association with the occurrence of diarrhea in foals, detection of the* fimH* gene simultaneously in strains recovered from feces and from urinary tract infections in other domestic animals [28] demonstrates the uropathogenic potential of fecal* E. coli* strains obtained from foals.

Two subunits of the* pap* gene in animals and humans have been extensively studied:* papC* and papG [28]. In the current study, the* papC* gene was detected in four diarrheic and in two nondiarrheic foals up to 90 days of age. These results demonstrate that this subunit of P fimbria (*papC*) has a little influence on enteric infections in foals. The* ag43* gene (antigen 43) has been recently demonstrated to encode an abundant outer membrane protein in* E. coli* [55]. Despite its unclear role as a virulence factor for* E. coli* in domestic animals,* ag43* expression has been implicated in chronic urinary tract infections in humans [56]. To the best of our knowledge, this study is the first to report identification of the* ag43* gene among* E. coli* strains isolated from diarrheic and nondiarrheic neonatal foals.

The* E. coli* O157:H7 serotype is an emergent causative agent of colitis and hemorrhagic uremic syndrome in humans, which are particularly fatal in children [18]. Stx or verocytotoxin is recognized as a major virulence factor of EHEC strains. In addition to Stx, enterohemorrhagic isolates contain the LEE pathogenicity island, which encodes intimin (*eae* gene) [57]. In the present study, none of 32* E. coli* strains expressed verocytotoxins (*vt1* and* vt2* genes) or* eae.* Furthermore,* ipaH* (EIEC), and* eae*,* eaf*, and* bfp* (EPEC), which are intimately associated with other pathotypes involved in human infections, were not identified in the 32* E. coli* strains isolated from our diarrheic and nondiarrheic foals. This circumstantial evidence indicates that foals from the studied region are not important carriers or reservoirs of the* E. coli* serotype O157:H7.

*R. equi* strains were isolated from three and six animals with and without diarrhea, respectively. Virulent* R. equi* from foals in the Americas, Japan, Australia, Africa, and Korea are predominantly VapA-expressing plasmid isolates, and they contain the 87-kb type I or 85-kb type I plasmid. The 85-kb type II plasmid has been observed in animals in France, whereas the 85-kb III and IV plasmids have been encountered in animals in the USA [55, 58–60]. Another finding with relevance in the current study was the detection of three VapA-positive* R. equi* strains in diarrheic foals between 30 and 60 days of age; two of these strains had the 85-kb type I and 87-kb type I plasmids. Interestingly, the same virulent plasmid types have been previously described in 41* R. equi* strains isolated from foals in Brazil; 33 of these strains had the 87-kb type I plasmid, and six contained the 85-kb type I plasmid, whereas the remaining two possessed a proposed new variant [59]. Our results confirm that the VapA 85-kb type I and 87-kb type I plasmids are most frequently present in virulent* R. equi* strains in foals from Brazil, and they reinforce the role of feces as a route of elimination of pathogenic* R. equi* in foals. In addition, to the best of our knowledge, this is the first report of detection of VapA 85-kb type I and 87-kb type I strains in diarrheic foals in South America.

Species from the Enterobacteriaceae family, such as* Hafnia alvei*,* Proteus mirabilis*,* Proteus vulgaris*,* Enterobacter agglomerans*,* Providencia rettgeri*, and* Citrobacter* sp., were identified in pure cultures from the diarrheic foals in this study. However, these microorganisms are rarely incriminated as primary causative agents of diarrhea in livestock, including foals. This finding reinforces the complexity of agents involved in enteric infections in foals [4, 52].

*Lawsonia intracellularis* was not detected by nested PCR in any of the fecal samples. The lack of identification of this pathogen in fecal samples from foals has been previously described [5, 7]. It was probably not detected in this study because only foals up to 90 days of age were analyzed, and* L. intracellularis* is prevalent in foals over six months of age [61].

Coronaviruses are infectious viruses that affect a wide range of domestic animals, wildlife species, and humans and cause a variety of gastrointestinal, respiratory, and occasionally neurologic symptoms. However, the real impact of this RNA virus as a primary causative agent of diarrhea in foals remains unclear [52]. In a study of enteropathogens in neonatal foals with and without diarrhea in Trinidad, the prevalence of coronaviruses was only 3% in the nondiarrheic group [13]. In the current study, only two (5.2%) foals were positive for coronaviruses: one diarrheic and one nondiarrheic animal. This very low prevalence indicates that coronaviruses likely have a minor influence on enteric infections in neonatal foals in the studied region.

Rotavirus is another enteric pathogen of neonatal foals with widely variable infection rates among different countries, and it has been reported in isolated cases and outbreaks and occasionally in association with a high mortality rate [7, 10, 13, 62]. Nevertheless, considering the large amount of this virus shed in feces and its viability in the farm environment, the potential for a rotavirus outbreak should be epidemiologically considered [63]. In the current study, none of the foals were positive for rotavirus. This result suggests that rotavirus has a low impact as a causative agent of diarrhea in neonatal foals in the studied region, similar to coronavirus.

Several reports from around the world have indicated that* C. parvum* is an important gregarine, within the subclass Cryptogregaria, agent of foal diarrhea [8, 9, 64, 65].* C. parvum* has been identified at a high frequency (25%) in diarrheic foals in Trinidad, although a significantly higher frequency has been detected in nondiarrheic animals [13]. In contrast, only 4% of the foals sampled in our study tested positive for* C. parvum*, including three with diarrhea and two without enteric signs. Another enteric protozoan pathogen,* Giardia duodenalis* (syn.* Giardia intestinalis*,* Giardia lamblia*), assemblage A, is considered the causative agent of diarrhea in many companion animals, livestock, wildlife species, and humans [18, 65]. However, few studies have investigated enteric infections by* Giardia intestinalis* and other parasite species in equines [52]. Similar to* C. parvum*,* G. duodenalis* was identified by molecular techniques in a small proportion (2%) of the analyzed stool samples from the diarrheic and nondiarrheic foals in the current study, indicating the limited involvement of this protozoan pathogen in enteric infections in the studied region.

*Strongyloides westeri* has been described as an important causative agent of neonatal foal diarrhea [1]. Interestingly, its prevalence (25%) was the same in the diarrheic and nondiarrheic foals in the present study. High prevalence rates (up 35%) of* S. westeri* in neonatal foals have been reported previously, with no significant differences between diarrheic and nondiarrheic animals [13]. In contrast, a low prevalence of* S. westeri* has been described in the United Kingdom [48]. In the present study, 15% of the foals tested positive for* Strongylus*. The higher frequencies of* S. westeri* and* Strongylus* across the studied farms probably partly reflect the inadequacy of deworming control programs.

### 4.1. Coinfections and Mortality Rates

Considerable variations in the frequencies of the major enteric pathogens recovered from diarrheic and nondiarrheic foals worldwide have been reported in different studies [1, 6, 7, 13]. Moreover, many studies have focused on only one enteric pathogen [8, 9, 9, 10, 12, 40, 63, 64], despite the recognized etiological complexity and the potential combinations of pathogens in equine enteric infections [13, 52]. Notably, a large number of enteric pathogens of bacterial, viral, and parasitic origins, as well as diverse coinfections, have been identified in diarrheic and nondiarrheic foals. In fact, substantial etiological complexity of enteric organisms has been observed among foals, reinforcing the necessity to include major enteropathogens in the differential diagnosis of neonatal enteric infections in these animals [1, 7, 17]. The failure to identify significant differences in the frequencies of pathogens between diarrheic and nondiarrheic foals in the current study, with the exception of* Clostridium perfringens* toxin A+, has also been reported previously [6, 66], indicating that other factors, such as age, the nutrition and immune statuses, environmental conditions, general management practices, and characteristics of the farm facilities, may influence the occurrence of clinical cases of diarrhea [4].

In addition, no significant associations of specific coinfections with the presence (or not) of diarrhea were detected, and a high mortality rate was observed among the* Salmonella*-positive diarrheic foals, regardless of the serotype or other pathogens present. High mortality of foals with enteric salmonellosis has also been described elsewhere [1, 66]. These results provide important information for practitioners who detect* Salmonella* spp. in the feces of foals because proper antimicrobial therapy, rigorous hydration, and other critical care measures are required to prevent serious complications and to improve prognosis.

## 5. Study Limitations

No clinical examinations or blood counts were carried out for the nondiarrheic foals; thus, the reference values for this species according the age were used.

Blood culturing and measurement of the serum IgG concentration were not performed, and necropsy and microbiological culturing of the enteric samples from the dead foals were not carried out due to the large number of included farms and because not all farms had veterinary residents.

Although the results related to virulence factors of* E. coli* were not statistically significant, their identification is important by increase of the understanding of the involvement of this pathogen in development of sepsis in foals.

Although we attempted to collect samples from diarrheic and nondiarrheic animals at all farms, it was not possible to collect samples from control animals (without diarrhea) at farms 4, 5, and 13.

The low prevalence related to the studied protozoa (*C. parvum* and* G. duodenalis*) may be related to intermittency of its release in the environment, as only one sampling time was used in this study for each foal.

## 6. Conclusions

A wide variety of enteric pathogens in foals were identified in this study. The frequency of* C. perfringens* toxin A+ significantly differed between the diarrheic and nondiarrheic animals, confirming that this toxin is a major virulence factor for enteric infections in foals.* Salmonella* spp. was the most prevalent pathogen associated with mortality among the diarrheic and nondiarrheic foals sampled, indicating the necessity of proper therapy and critical care of foals showing fecal isolation of this pathogen.

The detection of different serotypes of* Salmonella*, toxigenic* Clostridium perfringens* and* Clostridium difficile* strains, virulent* Rhodococcus equi* (VapA-positive), and* Cryptosporidium parvum* in feces from the diarrheic and nondiarrheic foals highlights a public health concern and indicates that practitioners should be careful when handling fecal material from foals because these organisms are recognized as human pathogens through fecal contamination of food and water.

## Figures and Tables

**Table 1:** Distribution of total foals per farm; sampled foals with (56) and without diarrhea (60) per farms, range of age, and number of dead foals.

Farm	Total foals/farm	Diarrheic foals	Nondiarrheic Foals	Age, days	Dead foals
	*N*	*N*	*N*	(range)	*N* (%)
1	10	7	3	(12–90)	ND
2	14	7	6	(24–90)	ND
3	10	1	3	(60–90)	ND
4	14	2	0	5 and 60	ND
5	U	1^†^	0	9	1
6	U	1	2	(68–90)	ND
7	40	1	1	1 and 25	ND
8	60	7^†^	1	(30–90)	1 (7)
9	30	2^†^	1^†^	(60–90)	2 (7)
10	12	2^††^	1^†^	(40–90)	3 (25)
11	22	7	9	(19–90)	ND
12	62	9	19	(1–90)	ND
13	10	3	0	(60–90)	ND
14	11	3^†^	4	(1–60)	1 (9)
15	20	0	10	(21–90)	ND

Total		**56**	**60**		**8**

*N* = number of foals; ND = nondetected; *N* (%) = number of dead foals/farm; U = uninformed; † = one dead foal; †† = two dead foals.

**Table 2:** Distribution of foals with (56) and without (60) diarrhea per farm and hematological and blood gas findings from diarrheic and nondiarrheic foals until 90 days of age. State of São Paulo, Brazil, 2011-2012.

Parameters (*n*)	Diarrheic foals (media)	Nondiarrheic foals (media)
Time of evaluation (days)	8.1 (±20.4)	0
HR (bpm)	87.6 (±24.1)	85.7 (±30.6)
RR (mpm)	41.8 (±20.3)	42.0 (±18.8)
Temperature (°C)	38.5 (±1.5)	38.7 (±0.6)

Parameters (*n*)	Diarrheic foals	^*∗*^Reference values

Erythrocytes ×10^6^/*µ*L (40)	9.3 (±1.6)	7.9–12.0
Hemoglobin g/dL (45)	10.0 (±4.8)	10.9–15.3
Hematocrit % (46)	35.2 (±5.9)	33.0–48.0
PT (plasma) g/dL	6.8 (±0.9)	4.3–7.0
Fibrinogen (mg/dL)	492.1 (±237.5)	200–500
Leucocyte ×10^3^/*µ*L	10.1 (±4,973.1)	6.2–15.0
Metamyelocyte/*µ*L	75.0 (±69.1)	0
Band neutrophils /*µ*L	199.8 (±237.2)	0
Segmented neutrophils/*µ*L	4.3 (±4,987.6)	4.1–5.4
Lymphocytes ×10^3^/*µ*L	1.8 (±1,728.9)	1–4.9
Eosinophils/*µ*L	87.5 (±148.1)	0–0.55
Basophils/*µ*L	64.0 (±77.8)	0–0.07
Monocytes ×10^3^/*µ*L	0.5 (±633.4)	0.07–0.76

pH	7.3 (±0.1)	7.32–7.44
PCO_2_ (mm Hg)	41.4 (±5.5)	52.7–114.2
PO_2_ (mm Hg)	35.8 (±9.1)	15.0–42.0
BE (mmol/L)	−2.1 (±7.5)	−7.33–3.9
HCO_3_ (mmol/L)	22.8 (±5.4)	19.0–27.4
tCO_2_ (mm Hg)	24.5 (±6.4)	31.0–33.0
SO_2_ (%)	59.4 (±11.7)	0
Na (mmol/L)	133.4 (±7.3)	148.0 ± 12
K (mmol/L)	3.1 (±0.7)	4.8 ± 1.0
iCa (mmol/L)	1.3 (±0.3)	0.68 ± 0.6

^*∗*^Reference values: Orsini, J. A.; Divers, T. J. Equine Emergencies. 3 ed. Elsevier, 2014. *n* = number of evaluated animals; CR = cardiac rate; RR = respiratory rate; bpm = beats per minute; mpm = respiratory movements per minute.

**Table 3:** Frequency of enteric pathogens and detection of selected virulence factors in diarrheic (*n* = 56) and nondiarrheic (*n* = 60) foals at three different ages. State of São Paulo, Brazil, 2011-2012.

Enteric pathogens	Age (days)							
	GI = 0–30 (*n* = 29)		GII = 31–60 (*n* = 30)		GIII = 61–90 (*n* = 57)		*N* = 116	*p* value
	Diarrheic	Nondiarrheic	Diarrheic	Nondiarrheic	Diarrheic	Nondiarrheic	Total	
	*n* (%)	*n* (%)	*n* (%)	*n* (%)	*n* (%)	*n* (%)	*n* (%)	
*Escherichia coli*	8 (27,58)	2 (6.89)	4 (13.33)	5 (16.66)	5 (8.77)	8 (14.03)	32 (27.58)	0.143
*Escherichia coli fimH*	4 (13.79)	4 (13.79)	8 (26.66)	2 (6.66)	5 (8.77)	9 (15.78)	32 (27.58)	0.097
*Escherichia coli ag43*	2 (6.89)	4 (13.79)	7 (23.33)	2 (6.66)	2 (3.50)	15 (26.31)	32 (27.58)	0.001
*Escherichia coli papC*	1 (3.44)	0 (0.0)	1 (3.33)	0 (0.0)	2 (3.50)	2 (3.50)	6 (5.17)	1.000

*Salmonella *Saintpaul	1 (3.44)	0 (0.0)	1 (3.33)	0 (0.0)	0 (0.0)	1 (1.75)	3 (2.58)	1.000
*Salmonella *Panama	1 (3.44)	0 (0.0)	0 (0.0)	0 (0.0)	1 (1.75)	0 (0.0)	2 (1.72)	*∗*
*Salmonella *Infantis	0 (0.0)	0 (0.0)	3 (10.0)	1 (0.0)	2 (3.50)	0 (0.0)	6 (5.17)	*∗*
*Salmonella *Typhimurium	0 (0.0)	0 (0.0)	0 (0.0)	0 (0.0)	3 (5.26)	1 (1.75)	4 (3.44)	*∗*
*Salmonella *enterica subsp. enterica	0 (0.0)	0 (0.0)	1 (3.33)	0 (0.0)	0 (0.0)	0 (0.0)	1 (0.86)	*∗*
*S*almonella Newport	0 (0.0)	0 (0.0)	0 (0.0)	0 (0.0)	0 (0.0)	1 (1.75)	1 (0.86)	*∗*
*Salmonella *Muenchen	0 (0.0)	0 (0.0)	0 (0.0)	0 (0.0)	1 (1.75)	0 (0.0)	1 (0.86)	*∗*

*Clostridium perfringens*	7 (24.13)	2 (6.89)	4 (13.33)	2 (6.66)	1 (1.75)	2 (3.50)	18 (13.79)	0.365
*C. perfringens *type A	7 (24.13)	2 (6.89)	4 (13.33)	0 (0.0)	1 (1.75)	4 (7.01)	18 (15.51)	0.033
*C. perfringens *type A *cpb2*+	1 (3.44)	0 (00.00)	0 (0.0)	2 (6.66)	0 (0.0)	1 (1.75)	4 (3.44)	0.500

*Clostridium difficile *infection (CDI)	1 (3.44)	0 (0.0)	0 (0.0)	0 (0.0)	0 (0.0)	0 (0.0)	1 (0.86)	0.500
A/B toxin detection	1 (3.44)	0 (0.0)	0 (0.0)	0 (0.0)	0 (0.0)	0 (0.0)	1 (0.86)	0.500
Toxigenic *C. difficile*	2 (6.89)	0 (0.0)	0 (0.0)	0 (0.0)	0 (0.0)	0 (0.0)	2 (1.72)	*∗*

*Rhodococcus equi*	0 (0.0)	2 (6.89)	0 (0.0)	1 (3.33)	0 (0.0)	2 (13.50)	5 (4.31)	*∗*
*R. equi* vapA 85 kb I	1 (3.44)	0 (0.0)	1 (3.33)	0 (0.0)	0 (0.0)	0 (0.0)	2 (1.72)	*∗*
*R. equi* vapA 87 kb II	1 (3.44)	0 (0.0)	0 (0.0)	1 (3.33)	0 (0.0)	0 (0.0)	2 (1.72)	*∗*

*Lawsonia intracellularis*	0 (0.0)	0 (0.0)	0 (0.0)	0 (0.0)	0 (0.0)	0 (0.0)	0 (0.0)	*∗*
Coronavirus	1 (3.44)	0 (0.0)	0 (0.0)	0 (0.0)	0 (0.0)	1 (1.75)	2 (1.72)	1.000
Rotavirus	0 (0.0)	0 (0.0)	0 (0.0)	0 (0.0)	0 (0.0)	0 (0.0)	0 (0.0)	*∗*
*Giardia duodenalis*	0 (0.0)	0 (0.0)	1 (3.33)	1 (3.33)	0 (0.0)	0 (0.0)	2 (1.72)	*∗*
*Cryptosporidium parvum*	1 (3.44)	1 (3.44)	1 (3.33)	1 (3.33)	1 (1.75)	0 (0.0)	5 (4.31)	1.000
strongylus	2 (6.89)	2 (6.89)	1 (3.33)	1 (3.33)	3 (5.26)	8 (14.03)	17 (14.65)	0.786
*Strongyloides westeri *	3 (10.34)	2 (6.89)	3 (10.0)	1 (3.33)	8 (14.03)	12 (21.05)	29 (25.00)	0.458

*N* = total of samples; *n* = number of positive samples; *∗* = no statistical difference; *C. perfringens* = *Clostridium perfringens; C. difficile* = *Clostridium difficile; R. equi* = *Rhodococcus equi; *G = group.

**Table 4:** Distribution of major enteric pathogens identified either isolated or combined (coinfections) among diarrheic and nondiarrheic foals until 90 days of age. State of São Paulo, Brazil, 2011-2012.

Enteric pathogens	Diarrheic (*n* = 56)		Nondiarrheic (*n* = 60)	
	*n*	(%)	*N*	(%)
Monoinfection	23	(41.07)	28	(46.67)
Coinfections	26	(46.43)	20	(33.33)
Negative^*∗*^	7	(12.50)	12	(20.00)

*n* = number of fecal samples; *∗* = isolation of nonconventional primary enteric pathogens of foals (*Hafnia alvei*, *Proteus mirabilis*, *Proteus vulgaris*, *Enterobacter agglomerans*, *Providencia rettgeri*, and *Citrobacter *spp.).

**Table 5:** Distribution of enteric pathogens identified as only one agent and combined infections from 56 diarrheic and 60 nondiarrheic foals. State of São Paulo, Brazil, 2011-2012.

Group	Type of infection	*E. coli*	*E. coli fimH*	*E. coli ag43*	*E. coli papC*	*Salmonella *Saintpaul	*Salmonella *Panama	*Salmonella *Infantis	*Salmonella *Typhimurium	*S. *enterica subsp. enterica
		*n* (%)	*n* (%)	*n* (%)	*n* (%)	*n* (%)	*n* (%)	*n* (%)	*n* (%)	*n* (%)
D	Monoinfection	10 (31.25)	8 (25.00)	6 (18.75)	1 (16.67)	1 (33.33)	0 (0.0)	0 (0.0)	1 (25.00)	1 (100.0)
	Coinfection	7 (21.88)	9 (28.13)	5 (15.63)	3 (50.00)	1 (33.33)	2 (100.0)	5 (83.33)	2 (50.00)	0 (0.0)
	Total number of isolates	17 (53.13)	17 (53.13)	11 (34.37)	4 (66.67)	2 (66.67)	2 (100.0)	5 (83.33)	3 (75.00)	1 (100.0)
ND	Monoinfection	9 (28.13)	6 (18.75)	9 (28.13)	1 (16.67)	0 (0.0)	0 (0.0)	0 (0.0)	0 (0.0)	0 (0.0)
	Coinfection	6 (18.75)	9 (28.13)	12 (37.50)	1 (16.67)	1 (33.33)	0 (0.0)	1 (16.67)	1 (25.00)	0 (0.0)
	Total number of isolates	15 (46.88)	15 (46.88)	21 (65.63)	2 (33.33)	1 (33.33)	0 (0.0)	1 (16.67)	1 (25.00)	0 (0.0)

		*S*. Newport	*S. *Muechen	*C. perfringens*	*C. perfringens *toxin A+	*C. perfringens *type A beta-2+	*C. difficile*	*C. difficile *tcdA+	*C. difficile *tcdB+	*R. equi*
		*n* (%)	*n* (%)	*n* (%)	*n* (%)	*n* (%)	*n* (%)	*n* (%)	*n* (%)	*n* (%)

D	Monoinfection	0 (0.0)	0 (0.0)	3 (16.67)	3 (16.67)	1 (25.00)	0 (0.0)	0 (0.0)	0 (0.0)	0 (0.0)
	Coinfection	0 (0.0)	1 (100.0)	9 (50.00)	9 (50.00)	0 (0.0)	1 (33.33)	0 (0.0)	0 (0.0)	0 (0.0)
	Total number of isolates	0 (0.0)	1 (100.0)	12 (66.67)	12 (66.67)	1 (25.00)	1 (33.33)	0 (0.0)	0 (0.0)	0 (0.0)
ND	Monoinfection	0 (0.0)	0 (0.0)	3 (16.67)	3 (16.67)	1 (25.00)	1 (33.33)	0 (0.0)	0 (0.0)	2 (40.00)
	Coinfection	1 (100.0)	0 (0.0)	3 (16.67)	3 (16.67)	2 (50.00)	1 (33.33)	1 (100.0)	1 (100.0)	3 (60.00)
	Total number of isolates	1 (100.0)	0 (0.0)	6 (33.33)	6 (33.33)	3 (75.00)	2 (66.67)	1 (100.0)	1 (100.0)	5 (100.0)

		*R. equi* vapA 85-kb I	*R. equi* vapA 87-kb I	*Lawsonia intracellularis*	Coronavirus	Rotavirus	*G. duodenalis*	*C. parvum*	Strongylus	*Strongyloides westeri*
		*n* (%)	*n* (%)	*n* (%)	*n* (%)	*n* (%)	*n* (%)	*n* (%)	*n* (%)	*n* (%)

D	Monoinfection	1 (50.00)	0 (0.0)	0 (0.0)	0 (0.0)	0 (0.0)	0 (0.0)	1 (20.00)	0 (0.0)	0 (0.0)
	Coinfection	1 (50.00)	1 (50.00)	0 (0.0)	1 (50.00)	0 (0.0)	1 (100.0)	2 (40.00)	6 (35.29)	14 (48.28)
	Total number of isolates	2 (100.0)	1 (50.00)	0 (0.0)	1 (50.00)	0 (0.0)	1 (100.0)	3 (60.00)	6 (35.29)	14 (48.28)
ND	Monoinfection	0 (0.0)	0 (0.0)	0 (0.0)	0 (0.0)	0 (0.0)	0 (0.0)	1 (20.00)	7 (41.18)	4 (13.79)
	Coinfection	0 (0.0)	1 (50.00)	0 (0.0)	1 (50.00)	0 (0.0)	1 (100.0)	1 (20.00)	4 (23.53)	11 (37.93)
	Total number of isolates	0 (0.0)	1 (50.00)	0 (0.0)	1 (50.00)	0 (0.0)	1 (100.0)	2 (40.00)	11 (64,71)	15 (51.72)

D = diarrheic foals; ND = nondiarrheic foals; *n* = total number of pathogen identifications; *E. coli* = *Escherichia coli*; *R. equi* = *Rhodococcus equi*; *C. parvum* = *Cryptosporidium parvum*; *G. duodenalis* = *Giardia duodenalis*; *C. perfringens* = *Clostridium perfringens*.

**Table 6:** Distribution of enteric coinfections from diarrheic (*n* = 6) and nondiarrheic (*n* = 2) dead foals. State of São Paulo, Brazil, 2011-2012.

	Enteric pathogens	Age (days)	Farm identification
Diarrheic foals	(i) *E. coli* + *C. perfringens* type A + *Salmonella* Panama + *Strongyloides westeri *+ strongylus	9	5
	(ii) *E. coli fimH* + *E. coli papC* + *Salmonella* Infantis + *Strongyloides westeri*	90	8
	(iii) *Salmonella* Typhimurium + *Strongyloides westeri*	90	9
	(iv) *Salmonella* enterica subsp enterica + *Strongyloides westeri* + strongylus	40	10
	(v) *Salmonella* Muechen + *Strongyloides westeri*	90	10
	ND	1	14

Nondiarrheic foals	(i) *E. coli* + *Salmonella* Infantis	60	9
	(ii) *E. coli fimH* + *E. coli ag43* + *Salmonella* Newport + *Strongyloides westeri*	90	10

NI = not identified.
